# Experience of Using a New Brain Surgery Head Frame and Location Sticker for Treating Spontaneous Intracranial Hematoma

**DOI:** 10.3389/fneur.2022.818523

**Published:** 2022-04-27

**Authors:** Hongyu Wang, Wenqiang Xin, Jianzhong Cui

**Affiliations:** ^1^Department of Surgery, Hebei Medical University, Shijiazhuang, China; ^2^Department of Neurosurgery, Tianjin Medical University General Hospital, Tianjin, China; ^3^Department of Neurosurgery, Tangshan Gongren Hospital, Tangshan, China

**Keywords:** intracerebral hemorrhage, brain surgery head frame, location sticker, evacuation, stroke

## Abstract

**Objectives:**

Various stereotactic aspirations have been accepted; however, no standard stereotactic aspiration has been established for the treatment of spontaneous intracerebral hemorrhage (ICH). The authors explored an easy, fast, and effective procedure by using a new brain surgery head frame and location sticker for the removal of spontaneous hematoma.

**Patients and Methods:**

A retrospective database review was performed from January 2018 to March 2020 to identify patients with ICH who were treated with puncture and drainage for hematoma by using a new brain surgery head frame and location sticker for positioning and guidance.

**Results:**

A total of 45 patients with spontaneous ICH were enrolled in our study. The mean (± *SD*) surgical time was 29.3 ± 4.1 min. The average hematoma evacuation rate was 72.2%. The mean (± SD) preoperative Glasgow Coma Scale (GCS) score was 9.58 ± 2.92; the mean GCS score increased to 11.55 ± 2.59 (*p* = 0.006) and 12.86 ± 2.04 (*p* < 0.001) at 1 week after surgery and at the time of discharge, respectively. The mean (± *SD*) preoperative muscle force score was 1.25 ± 1.51; the mean muscle force score had improved to 2.20 ± 1.64 (*p* = 0.009) and 2.88 ± 1.64 (*p* < 0.001) at 1 week after the operation and the time of discharge, respectively. Out of these, one patient experienced postoperative rebleeding, however, no further hematoma expansion was found after the second aspiration and thrombolysis.

**Conclusion:**

Using this brain surgery, head frame and location sticker combined with urokinase infusion appears simple, safe, and effective for the removal of hematoma for patients with spontaneous ICH. However, randomized controlled trials are necessary to provide more concrete evidence-based results.

## Introduction

Currently, intracerebral hemorrhage (ICH), a serious and common cerebrovascular disease, is associated with high mortality and adverse clinical outcomes, especially in the elderly (1). Around 10% of strokes and 30% of cerebrovascular diseases are caused by ICH, which affects ~4,000,000 people per year worldwide, with 40% mortality at 1 month (2–4). Meanwhile, 40% of survivors are severely disabled, and only 12% of them can live independently, having a huge impact on families and society (5, 6). Surgery is the most common treatment of ICH, and it has been greatly improved with the accumulation of surgical experience and progress in scientific research (7). However, there are different surgical procedures with varying prognoses (8, 9). Craniotomy is the traditional surgical treatment, and good effects for hematoma removal and neurocognitive function improvement have been reported in many studies. However, craniotomy always damages normal brain tissues in the area around the hemorrhage, subsequently affecting the efficacy of craniotomy for the treatment of patients with ICH (10–12). Meanwhile, the appearance of minimally invasive endoscopic techniques and their promising application perspectives have been demonstrated (13). There are two primary kinds of minimally invasive surgery, such as endoscopic evacuation and stereotactic aspiration, for removing the hematoma. In endoscopic evacuation, the endoscope is placed into the hematoma by creating a small bone window, and suction and irrigation are then used to remove the hematoma. For stereotactic aspiration, a catheter is inserted into the center of the hematoma, and then the hematoma is suctioned using different image guidance. The catheter can also be left in the center of the hematoma to repeatedly instill thrombolytic drugs to prevent any residual hematoma. Currently, although various stereotactic aspirations have been accepted and show differing results, no standard stereotactic aspiration technique has been established for the treatment of spontaneous ICH (14). Here, we present our single-center experience with applying an easy, fast, and effective procedure for the removal of hematoma in 45 consecutive patients with spontaneous ICH.

## Materials and Methods

We conducted a retrospective study involving 45 patients treated from January 2018 to March 2020 at the Department of Neurosurgery, Tangshan Gongren Hospital. All the patients with ICH were treated with puncture and drainage for cerebral hematoma by using a new brain surgery head frame and location sticker for positioning and guidance. Computed tomography (CT) scans revealed that the volume of intracranial supratentorial hemorrhage was more than 20 ml and subtentorial hemorrhage was more than 10 ml. These patients required surgical treatment. The incidence of hematoma evacuation and rebleeding, operative time, Glasgow Coma Scale (GCS) score, and muscle force at 1 h preoperatively, 1 week postoperatively, and discharge time were analyzed.

### Ethics

This trial was approved by the Medical Ethical Committee of Tangshan Gongren Hospital. Signed consent forms were obtained from all the patients before the operation or from an immediate family member, if the patient was unable to do so.

### Inclusion and Exclusion Criteria

Patients were included in our study, if they met the following preplanned criteria: (I) diagnosed with spontaneous supratentorial hemorrhage from 20 to 50 ml or subtentorial hemorrhage from 10 to 15 ml; (II) age between 25 and 80 years; (III) spontaneous ICH occurring within 72 h; (IV) GCS score ≥ 5; and (V) normal coagulation function.

The exclusion criteria were as follows: (I) ICH caused by intracranial aneurysm, traumatic brain injuries, or cerebrovascular malformation; (II) coagulation dysfunction; (III) serious heart, renal, or lung functional failure; (IV) neurological deficits or cerebrovascular events before ICH; and (V) incomplete or missing consent form.

### Brain Surgery Head Frame and Location Sticker

**Figure 1:**
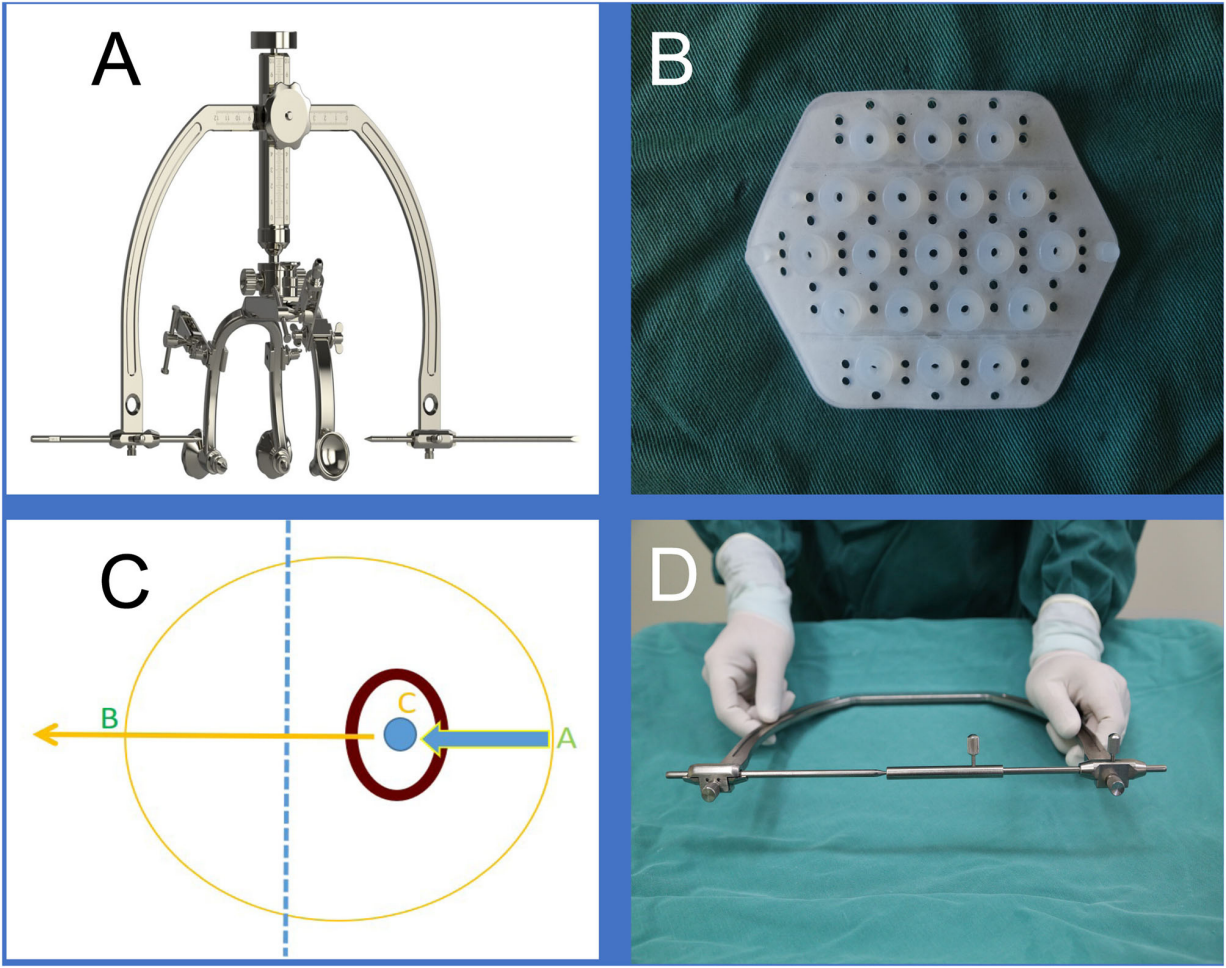
**(A)** is the brain surgery head frame, **(B)** is the location sticker, **(C and D)** shows that our design was based on the theory of “three points on one line,” where the three points are the scalp puncture site, the hematoma center site, and the reference point, and the one line refers to the straight line formed by the three points.

The brain surgery head frame (patent number: ZL 201621187091.5) and the location sticker (patent number: ZL 201720977420.4) are based on the theory of “three points on one line” (Figures 1A,B) The three points are the hematoma center site, scalp puncture point, and scalp reference point. One line refers to the straight line formed by these points, namely, the surgical puncture route (Figures 1C,D).

### Intervention

**Figure 2:**
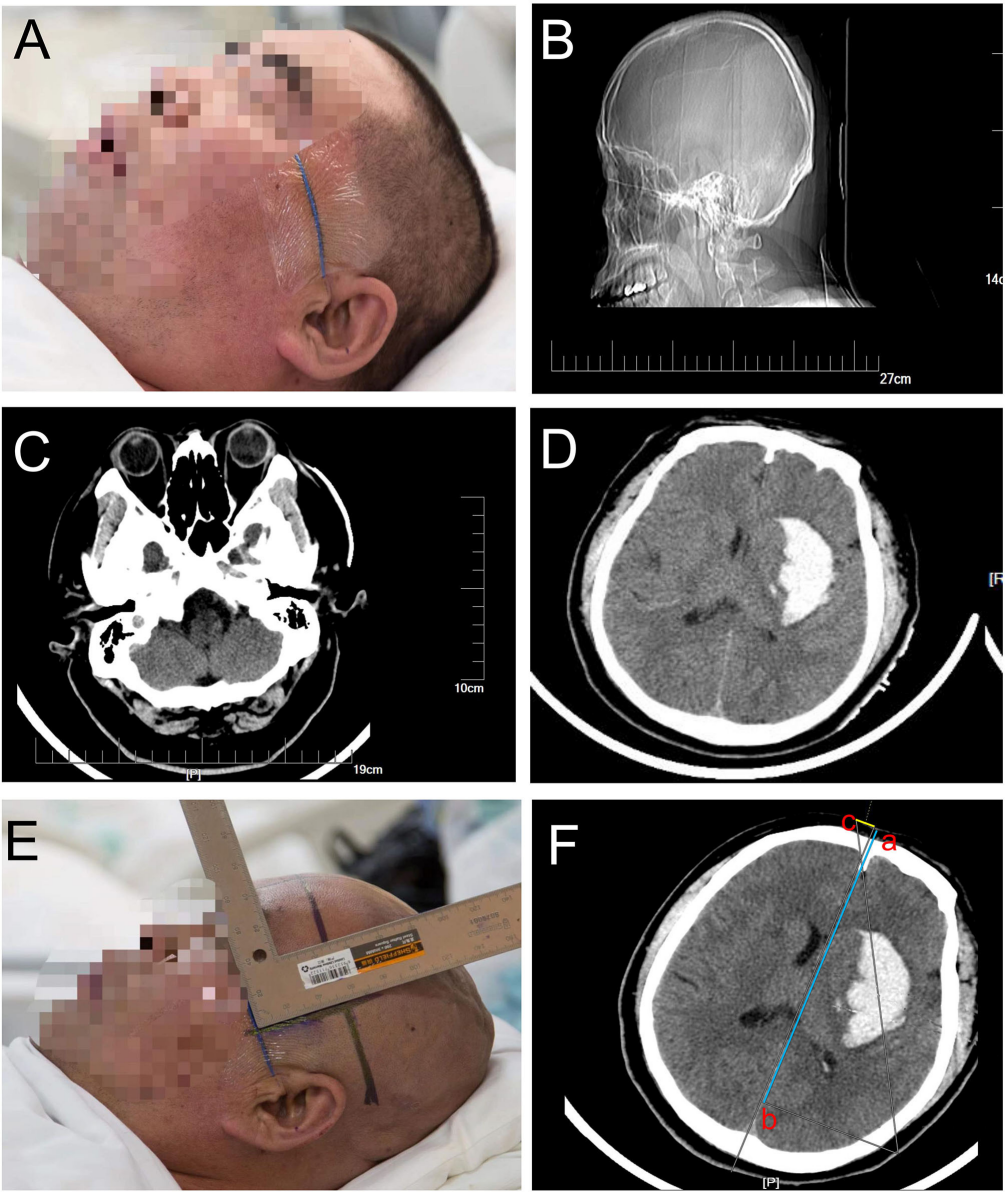
**(A)** shows that the orbitomeatal line is the preparation before the CT scan for patients with suspected cerebral hemorrhage, **(B)** confirms that the orbitomeatal line was precisely attached to the scalp, **(C)** represents the slice marked by the orbitomeatal line as the first slice of the CT scan, intracranial hemorrhage was confirmed in **(D)**, and the punctured plane was measured based on the distance between the first slice and the largest slice of the hematoma in **(E)**. **(F)** represents how to determine the position of the location stickers on the CT image by measuring and marking their positions on the scalp.

**Figure 3:**
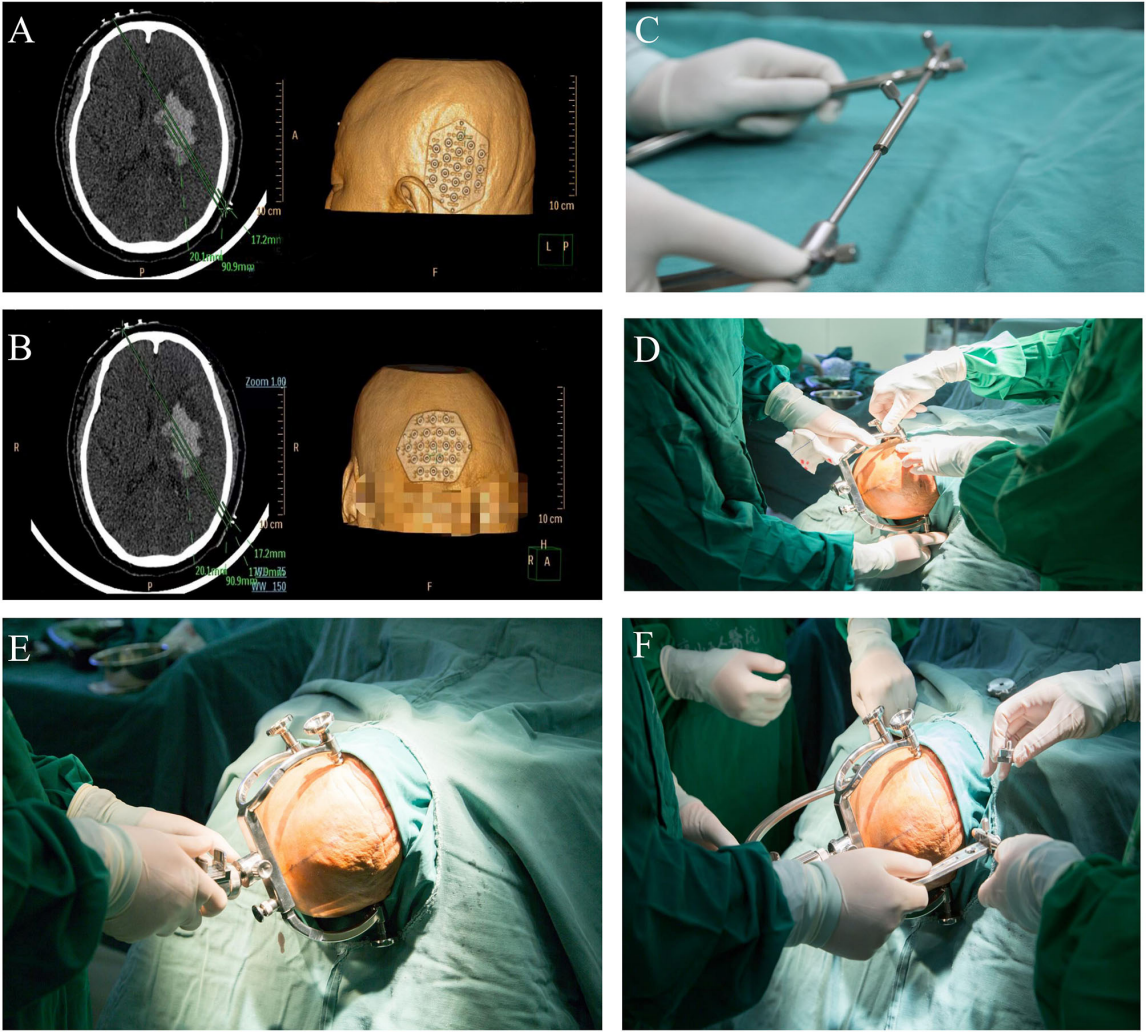
**(A,B)** show that the accurate puncture site, reference point, puncture route, puncture depth, and drill depth are located on the skin by performing a CT scan with 1 mm for each layer using the PISP (Philips IntelliSearch Portal v5.0.2.10010) system. The brain surgery head frame is correct in **(C)**, and **(D–F)** is the fixation process of the head frame.

**Figure 4:**
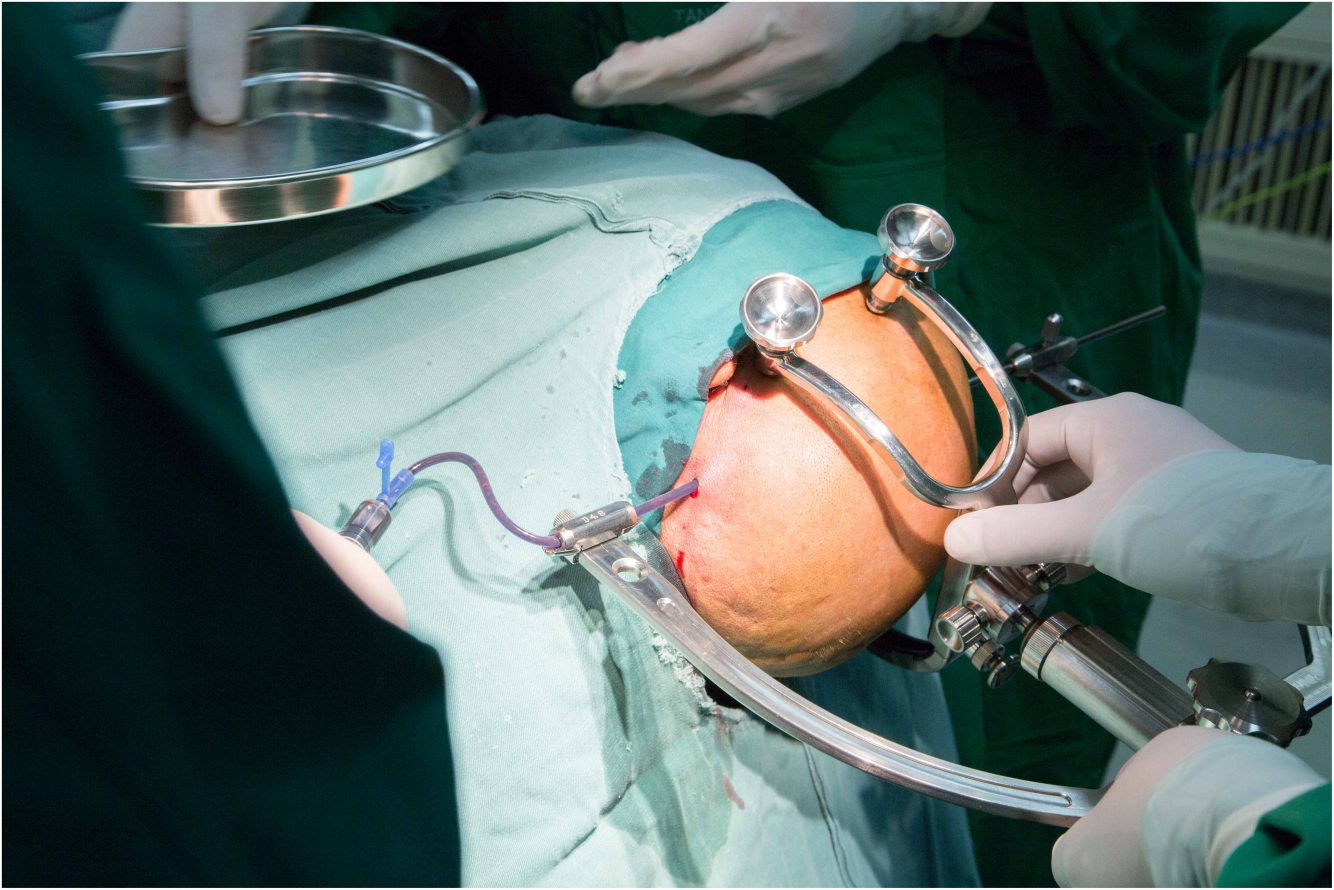
Puncture and aspiration of the hematoma.

Before admission to the operation room, all the patients underwent a CT scan to confirm the appearance of an intracranial hemorrhage requiring evacuation (Figure 2A). The waiting time before the operation in all the patients was set for at least 6 h after onset owing to most rebleeding appearing within 6 h after onset according to several publications (15–17). Then we made the preoperative surgical plans. Based on the location and volume of the intracranial hematoma observed on the preoperative CT scan, we roughly located the hematoma center site and puncture route on the largest slice of the hematoma seen on the CT image (Figures 2B–E) and then marked the puncture point and reference point of the hematoma on the scalp where the location sticker would be attached (Figure 2F). A CT scan with 1 mm for each layer was performed to locate an optimal slice of the hematoma to measure the precise drilling and puncture depth and to obtain a puncture point and reference point that would appear in the location sticker by using the PISP (Philips IntelliSearch Portal v5.0.2.10010 or v7.0.6.40181) system and then mark them on the scalp before surgery (Figures 3A,B). The best puncture route should generally meet the following conditions: (I) avoid the important functional areas and large blood vessels; (II) along the hematoma axis as much as possible; and (III) as short as possible. We adjusted the brain surgery head frame before it was fixed on the scalp to be perpendicular to the puncture path (Figures 3C–F). The two ends of the brain surgery head frame were accurately fixed at the puncture point and reference point to place the puncture point, reference point, and hematoma center point on the puncture path. Aspiration of the hematoma was conducted after drilling through the skull and arriving at the puncture site according to the preset depth on the second CT scan (Figure 4). If the resistance increased, the hematoma aspiration was stopped, and a drainage tube was inserted into the cavity of the hematoma combined with a urokinase drip for several days (50,000 IU every 8 h) in 3 ml of normal saline through the hematoma catheter to remove the remaining hematoma. If a multitarget puncture was required, we conducted the hematoma aspiration according to the method described above with several catheters.

Total intravenous anesthesia (TIVA) was used during the surgery. Anesthesia induction: intravenous midazolam 0.04–0.06 mg/kg, propofol 1.5–2.5 mg/kg, sufentanil 0.3–0.5 μg/kg, and rocuronium 0.6 mg/kg. Mechanical ventilation was performed after anesthesia induction and endotracheal intubation. The tidal volume was 6–8 ml/kg, the ventilation frequency was 12 times/min, the inhalation-exhalation ratio was 1.0:2.0, the inhalation oxygen concentration was 40–60%, and the oxygen flow was 2 L/min. The anesthesia maintenance drugs were relifentanil 0.1–0.3 μg/kg·min and propofol 4–6 mg/kg/h. Rocuronium bromide was intermittently given with a dosage of 0.15 mg/kg, bispectral index (BIS) was maintained between 40 and 60, and vasoactive drugs were given when necessary to maintain the blood pressure and heart rate within the normal range.

In addition, early intensive hypotension is safe and effective and can significantly improve patient outcomes. If there is a preoperative systolic blood pressure of 150–220 mm Hg and no contraindications for acute antihypertensive therapy, it should be reduced to <140 mm Hg based on a good cerebral perfusion. The target value of decompression for patients with more than 220 mmHg should be based on the patient's history of hypertension and basic blood pressure, but it can be reduced to below 160 mmHg. The intraoperative and postoperative blood pressure should be under 140 mmHg based on a good cerebral perfusion.

### Hematoma Volume Calculations

The hematoma volumes were calculated using the ABC/2 formula, where A and B are the perpendicular maximal diameters of the lesion, and C is the total length in the vertical plane [equation: V = (length × width × thickness)/2]. All diameters and lengths were obtained from the CT scans taken just before the operation (18).

### Imaging Follow-Up

Cranial CT was conducted 2 h after the drainage tube was removed, and the incidence of hematoma evacuation was calculated by the same person blinded to all treatment and outcomes using Slicer software (19).

### Statistical Analysis

We used SPSS statistical software (version 19.0, IBM Corp.) to conduct our analyses, and a *p* < 0.05 was considered to reveal a significant difference. We used the paired Wilcoxon test to compare the preoperative hematoma volume and time of tube removal. For patients with supratentorial ICH, the Kruskal–Wallis test was used to compare the preoperative initial GCS score, GCS score at 1 week after the operation, and discharge time, and multiple comparisons in one-way analysis of variance (ANOVA) were used to compare the preoperational muscle power, 1 week after the operation, and at the discharge time.

## Results

Total forty-five patients with spontaneous ICH were enrolled in our study, including 30 cases of putamen hemorrhage, 5 cases of thalamic hemorrhage, 5 cases of lobar hemorrhage, and 5 cases of cerebellar hemorrhage. The mean age of the patients was 57.13 ± 10.29 years, and there were 33 men and 12 women. Out of these, thirty-one patients (71.11%) had a prior history of hypertension, and 26 were being treated with medications. More details of the patients' clinical and demographic characteristics are shown in Table 1. The brain surgery head frame combined with a location sticker was used to ensure that the optimal puncture was made and that the soft drainage tube reached the center of the hematoma in all cases. Although not all hematomas were initially removed, all patients eventually achieved satisfactory removal of the hematoma.

**Table 1:** Demographic and clinical characteristics of the 45 patients in the study.

**Case No**.	**Age**	**Gender**	**H, D, or C History**	**MH**	**Side**	**Location**	**Pre mRS**	**3 M** ** mRS**	**Six M mRS**	**Pre** ** GOS**	**Six M GOS**	**TSOHA**	**Vol(ml)**	**Rv(ml)**	**ICD**	**Operation Time**	**LOS**
1	57	male	H&C	No	Left	Putamen	4	3	3	3	4	13	25.6	5.8	3	27 mins	20
2	48	male	H&D	No	Left	Putamen	4	2	2	3	5	12	32	6	3	26 mins	25
3	67	male	H&D	Anti-P	Left	Parietooccipital	4	3	3	3	4	10	48.36	7.2	3	35 mins	25
4	42	female	H	No	Left	Putamen	4	2	2	3	5	13	37.4	4.5	2	27 mins	16
5	54	male	H	No	Right	Putamen	4	2	1	3	5	8	31.5	3.5	2	28 mins	18
6	52	male	H	No	Right	Putamen	4	3	3	3	4	9	30.7	8	4	27 mins	17
7	49	male	H	No	Left	Temporoparietal	3	2	2	4	5	8	31	8.5	4	29 mins	16
8	72	female	No	No	Left	Occipital	3	2	2	4	5	7	46	6.7	4	28 mins	30
9	47	male	H	No	Left	cerebellum	4	2	2	3	5	8	12.3	3.18	2	26 mins	18
10	76	female	H	No	Left	Putamen	5	4	4	2	3	10	46	11.3	2	35 mins	35
11	39	male	H	No	Left	Thalamus	5	3	3	2	4	8	23.6	3.6	3	27 mins	21
12	51	male	No	No	Left	Putamen	4	3	3	3	4	8	28.5	4.6	3	25 mins	23
13	42	male	H	No	Right	Putamen	4	3	3	3	4	8	39.5	6.6	3	28 mins	24
14	65	male	H	No	Right	Putamen	5	4	4	2	3	9	48.9	4.6	4	37 mins	23
15	63	female	H	No	Left	Putamen	5	3	2	2	5	9	26.5	3.96	3	26 mins	18
16	56	male	H	No	Left	Putamen	5	4	4	2	3	10	46.6	9.7	2	35 mins	23
17	44	male	H	No	Left	Temporal	4	1	1	3	5	7	22	5	2	23 mins	18
18	55	male	H	No	Left	Putamen	5	2	1	2	5	16	31	7.4	4	27 mins	30
19	58	female	H	No	Left	Putamen	4	3	3	3	4	10	26	4.27	3	27 mins	18
20	51	male	H	No	Left	Putamen	4	3	3	3	4	7	32.5	6.8	3	30 mins	21
21	65	male	H	No	Right	cerebellum	3	2	2	4	5	8	13.5	3.5	3	26 mins	18
22	60	female	H	No	Left	Thalamus	5	4	4	2	3	9	22.4	4.5	4	28 mins	21
23	77	male	H	No	Left	Putamen	5	4	4	2	3	8	45.6	8.6	4	34 mins	28
24	52	male	H	No	Left	Putamen	4	3	2	3	5	8	33.1	5.8	2	25mins	23
25	42	male	H	No	Left	Putamen	4	3	3	3	4	13	37.41	2.3	3	35 mins	22
26	51	male	H	No	Right	Putamen	4	3	2	3	5	12	44.5	5.7	3	37 mins	20
27	51	male	No	No	Left	Putamen	4	3	2	3	5	10	33.1	3.8	2	29 mins	18
28	45	male	H	No	Right	cerebellum	3	1	1	4	5	13	12.5	3.8	3	28 mins	21
29	54	male	No	No	Left	Putamen	4	3	3	3	4	8	29.5	5.3	3	25 mins	18
30	51	male	H	No	Right	Putamen	4	3	2	3	5	9	33.7	5.6	4	28 mins	22
31	67	male	H	No	Right	Temporoparietal	4	3	2	3	5	8	45.21	8.5	4	37 mins	25
32	56	male	H&D	No	Right	Putamen	5	4	4	2	3	7	33.9	7.7	3	26 mins	30
33	62	female	No	No	Left	Putamen	4	3	2	3	5	8	30.6	7.2	4	26 mins	18
34	70	female	H	No	Right	cerebellum	3	2	1	4	5	10	11.5	2.2	3	28 mins	20
35	50	female	No	No	Left	Putamen	4	3	3	3	4	8	43	8	4	33 mins	20
36	45	male	H&D	No	Right	Putamen	5	3	3	2	4	8	43.29	10.7	4	32 mins	28
37	71	male	No	No	Right	Putamen	5	4	4	2	3	8	44.2	9.8	3	37 mins	26
38	72	female	No	Anti-P	Left	Putamen	5	4	4	2	3	9	31.5	5	3	29 mins	18
39	50	female	H	Anti-P	Right	Thalamus	5	4	4	2	3	9	22.6	5.7	3	28 mins	30
40	65	male	H	No	Right	Putamen	4	3	3	3	4	10	46.6	10.8	3	35 mins	18
41	60	male	H&D	No	Right	cerebellum	4	2	2	3	5	7	13.1	3.8	3	25 mins	31
42	71	female	H	No	Right	Putamen	5	4	3	2	4	16	37.9	6.9	3	36 mins	27
43	69	male	H	No	Left	Thalamus	5	4	3	2	4	10	23.6	6.6	3	24 mins	35
44	55	male	H	No	Right	Putamen	4	3	3	3	4	7	31.7	5.64	2	27 mins	25
45	66	male	H	No	Right	Thalamus	5	4	4	2	3	8	22.4	5.3	3	28 mins	36

*ICD, Indwelling catheter days; TSOHA, time from symptom onset to hematoma aspiration; LOS, length of stay; Pre, Previous; H, hypertension; D, diabetes; C, coronary heart disease; MH, medication history; Anti-P, antiplatelet; M, months; mRS, Modified Rankin Scale; GOS, Glasgow Outcome Scale*.

**Figure 5:**
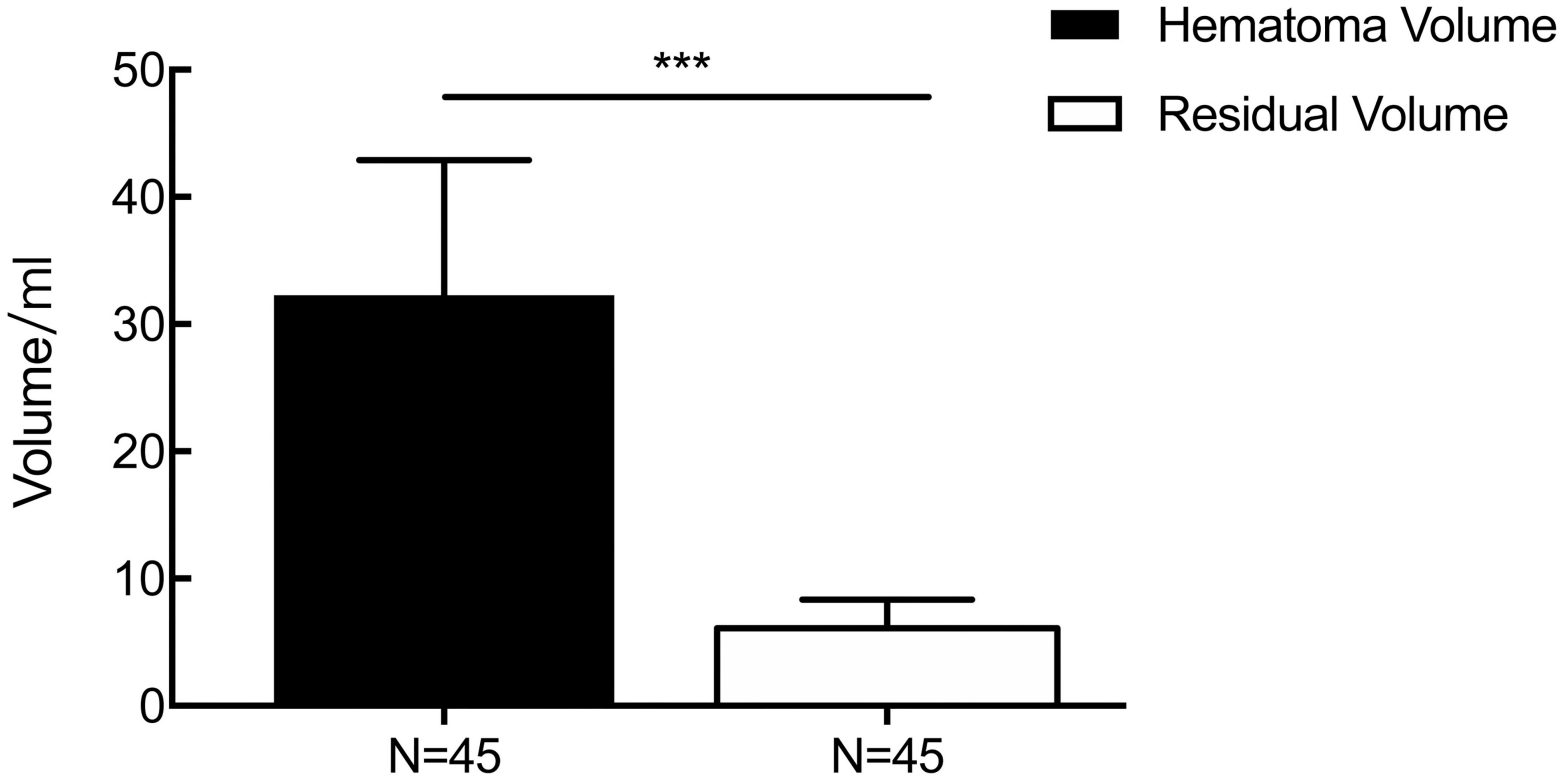
The result of changes in the volume of the hematoma perioperatively. The mean hematoma volume was 32.27 ± 10.63 ml before the operation, while the average left hematoma volume was 6.08 ± 2.27 ml until the drainage tube was removed (*p* < 0.001). ***p < 0.001.

The time from symptom onset to the first aspiration was more than 6 h in all cases. The mean hematoma volume was 32.27 ± 10.63 ml before the operation, while the average left hematoma volume was 6.08 ± 2.27 ml until the drainage tube was removed (*p* < 0.001, Figure 5), showing a mean evacuation rate of 72.20%. The soft drainage catheter was inserted for a median duration of 2.76 days.

**Figure 6:**
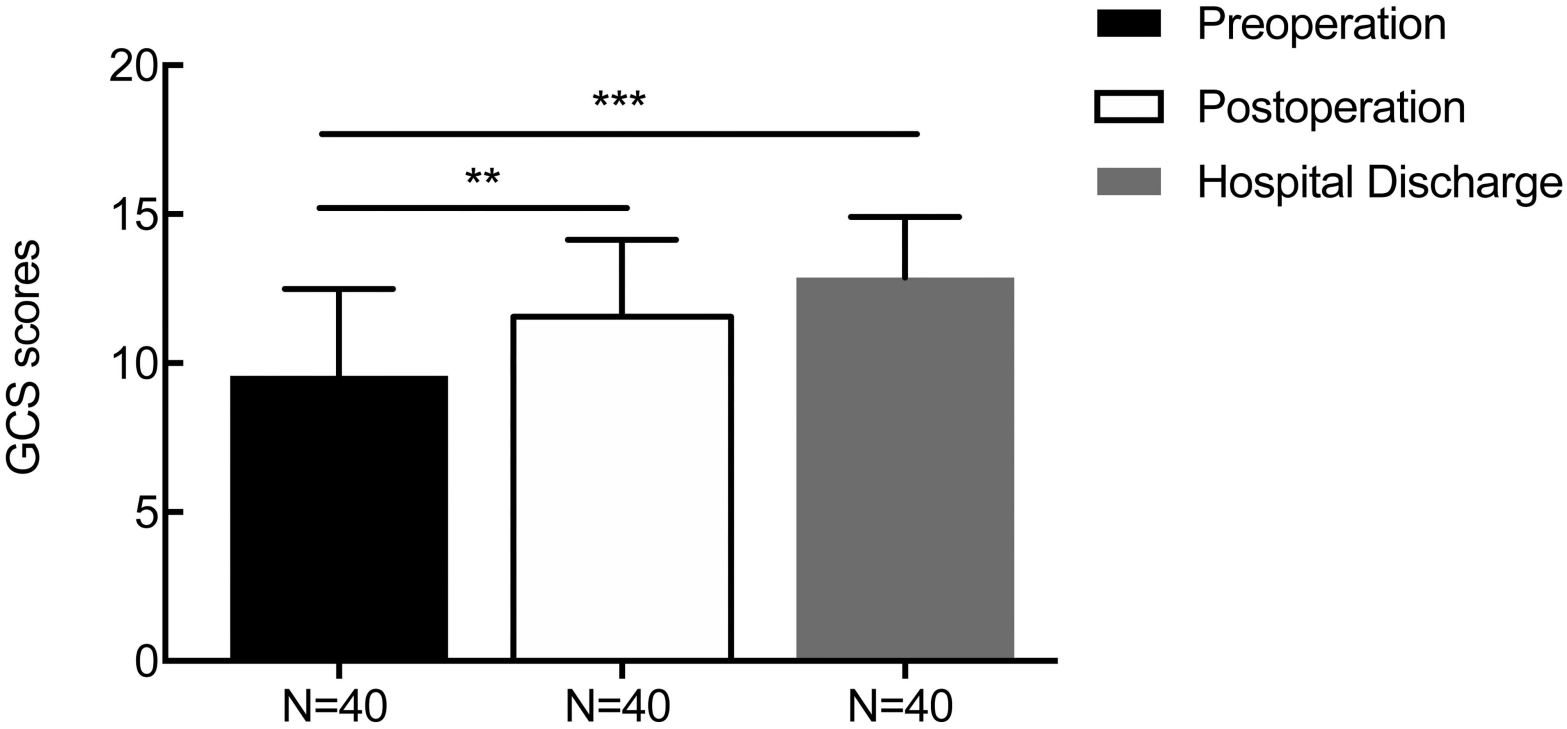
The result of changes in the perioperative GCS score. The average initial GCS score was 9.58 ± 2.92, and the mean GCS scores at 1 week after the operation and at discharge were 11.55 ± 2.59 (*p* = 0.006) and 12.86 ± 2.04 (*p* = 0.000), respectively. **p < 0.01 and ***p < 0.001.

**Figure 7:**
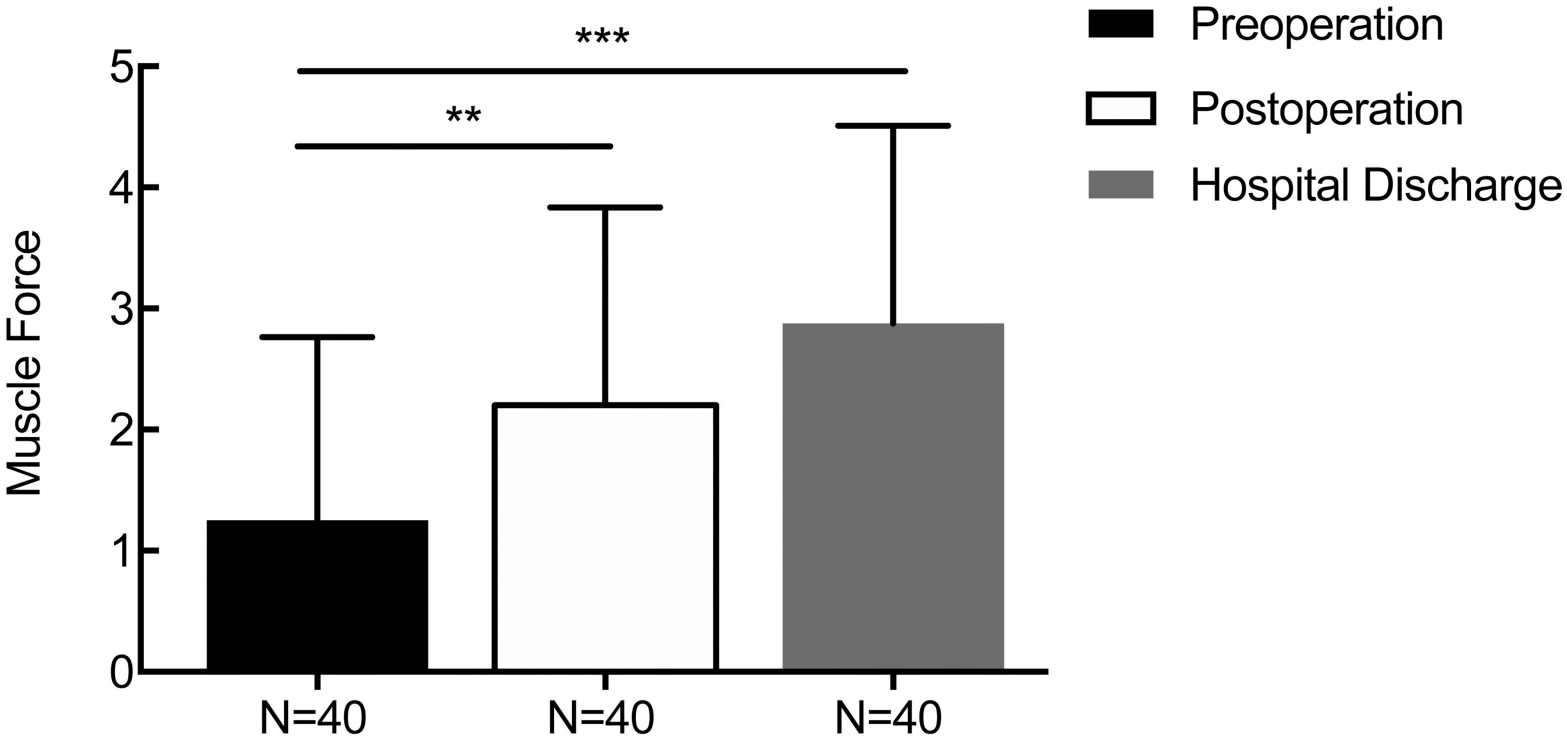
Changes in the perioperative muscle force score. The average preoperative muscle power was 1.25 ± 1.51, and the mean muscle power at 1 week after the operation and discharge time were 2.20 ± 1.64 (*p* = 0.009) and 2.88 ± 1.64 (*p* < 0.001), respectively. **p < 0.01 and ***p < 0.001.

For patients with supratentorial ICH, the average initial GCS score was 9.58 ± 2.92, and the mean GCS score at 1 week after the operation and discharge time were 11.55 ± 2.59 (*p* = 0.006) and 12.86 ± 2.04 (*p* = 0.000, Figure 6), respectively. The average preoperative muscle power was 1.25 ± 1.51, and the mean muscle power at 1 week after the operation and at the discharge time were 2.20 ± 1.64 (statistically significant improvement, *p* = 0.009) and 2.88 ± 1.64 (statistically significant improvement, *p* < 0.001, Figure 7), respectively. Meanwhile, mRS and GOS scores were assessed preoperatively and at 6 months postoperatively. The results showed that the majority of patients improved significantly in neurological function based on the mRS (mean 4.244 reduced to mean 2.689, *p* < 0.001) and GOS scores (man 2.765 increased to mean 4.200, *p* < 0.001). More details were shown on Table 1.

General anesthesia was used for all cases. The mean (± *SD*) surgical time was 29.3 ± 4.1 min. Preoperative surgical plans, such as designing an accurate puncture site, reference point, puncture route, puncture depth, and drill depth data processing, were completed within ~5 min, and the median time from the initial head frame fixation to its removal was 20–30 min for the patients needing a single tube for aspiration, while it was 30–40 min for patients needing two tubes. There was no intracranial infection or systemic hemorrhage in any of the patients. No patient died before being discharged from the hospital. Although there was one patient who experienced postoperative rebleeding, no further hematoma expansion was found after the second aspiration and thrombolysis.

### Illustrative Cases

#### Case 1: A Cerebellar Hemorrhage

**Figure 8:**
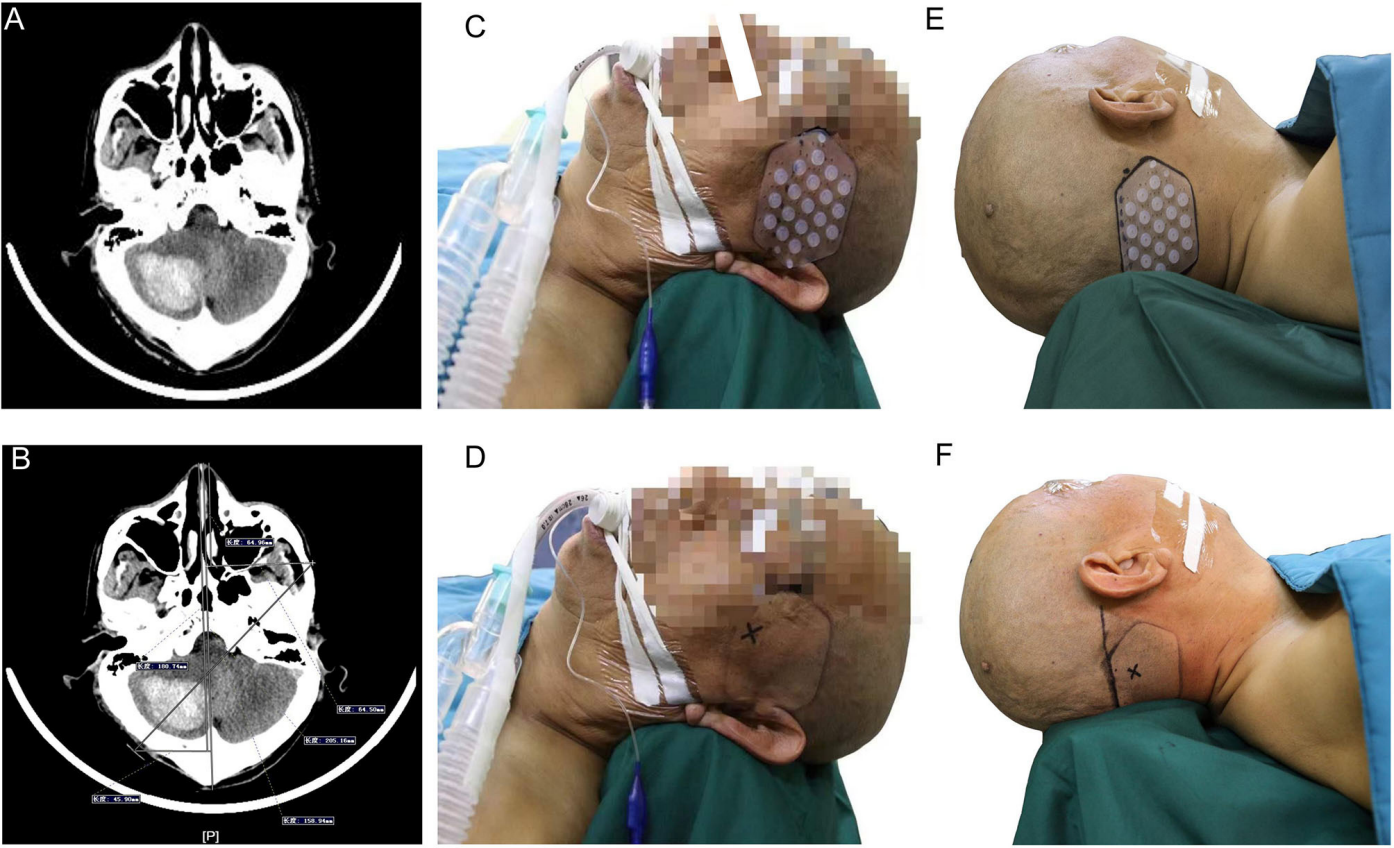
Right cerebellar hemorrhage shown in **(A)**. A vertical distance of 45.90 mm (puncture point) from the centerline and a parallel distance of 64.96 mm (reference point) along the centerline are shown in **(B)** to locate the location sticker in **(C–F)**.

**Figure 9:**
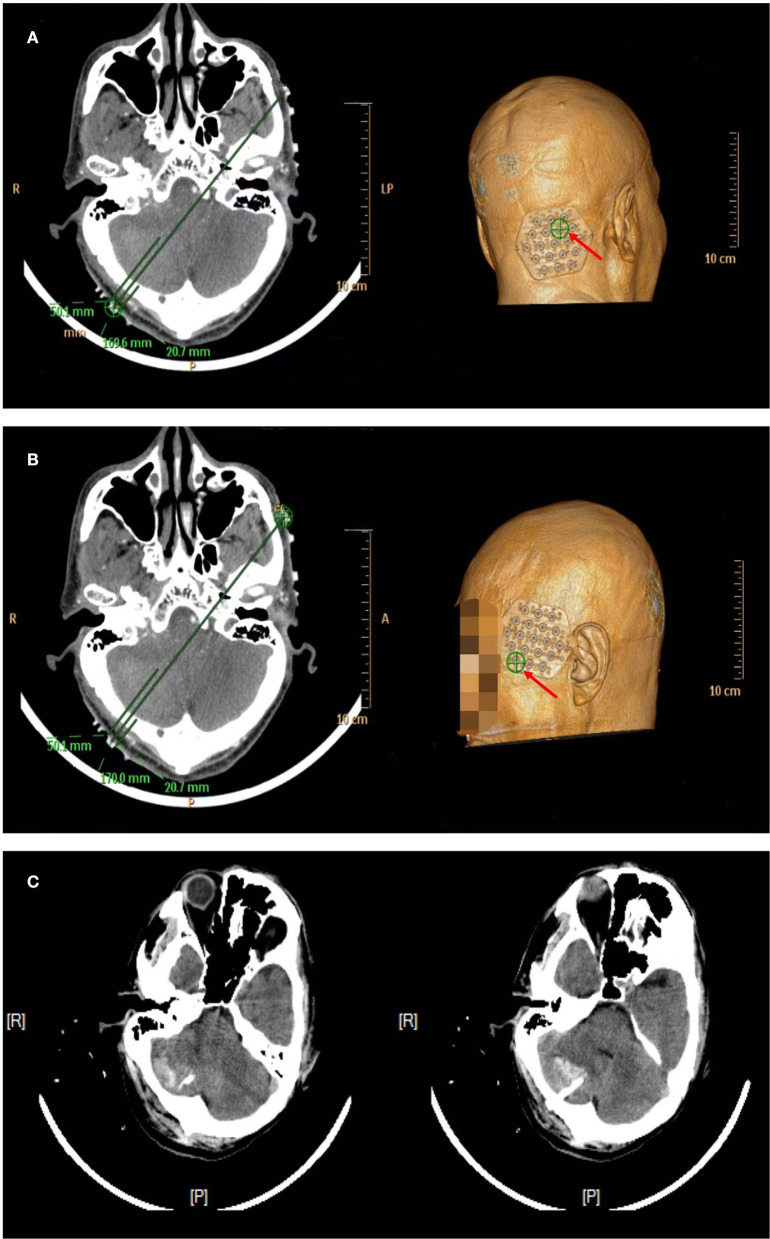
The accurate puncture site, reference point, puncture route, puncture depth (50.1 mm), and drill depth (20.7 mm) are shown in **(A,B)** when using the PISP system. **(C)** shows the good clinical outcomes.

A 65-year-old man with a history of 2 years of hypertension was admitted to our hospital for sudden headache, dizziness, and vomiting. Cranial CT revealed a right cerebellar hemorrhage (Figure 8A). An optimal image for attaching the patches was available based on the distance between the orbitomeatal line and the largest area of the hematoma seen on the CT image. Based on the vertical distance of 45.90 mm (puncture point) from the centerline and a parallel distance of 64.96 mm (reference point) along the centerline (Figure 8B), we achieved a location sticker on the patient's head (Figures 8C–F). We performed a CT scan of 1 mm for each layer to reveal the accurate puncture site, reference point, puncture route, puncture depth (50.1 mm), and drill depth (20.7 mm) by using the PISP (Philips IntelliSearch Portal v7.0.6.40181) system before surgery (Figures 9A,B). We fixed the positioning and guided the brain surgery head frame on the head perpendicular to the puncture trajectory, and then 5 ml hemorrhage was suctioned by using a drainage tube inserted in the hemorrhage for 3 days combined with urokinase administration. The cranial CT conducted the next day demonstrated close to complete evacuation of the hematoma (Figure 9C).

#### Case 2: A Basal Ganglia Hemorrhage

**Figure 10:**
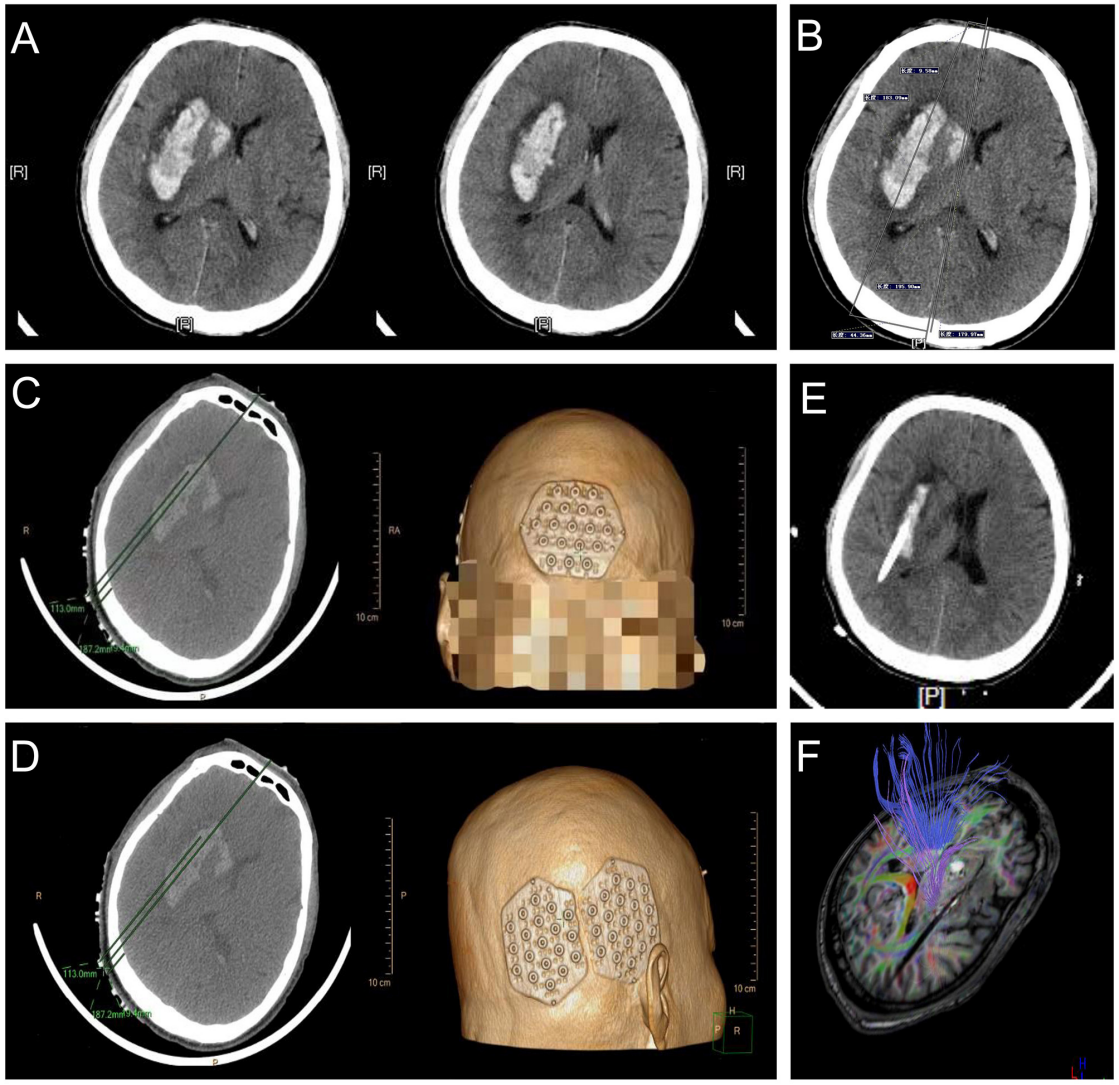
Basal ganglia hemorrhage appears in **(A)**, with a vertical distance of 9.58 mm (reference point) from the centerline and a parallel distance of 179.97 mm (puncture point) along the centerline in **(B)** used to locate the location sticker in **(C,D)**. **(E,F)** represent good clinical outcomes.

A 54-year-old man with a history of 5 years of hypertension was admitted to our center after a sudden confusion of consciousness and left limb paralysis. Emergency brain CT scans showed a right basal ganglia hemorrhage (Figure 10A). The distance between the orbitomeatal line and an optimal section of the hematoma shown in the CT image was 4.5 cm. Two location stickers were attached on the scalp at a vertical distance of 9.58 mm (reference point) from the centerline and a parallel distance of 179.97 mm (puncture point) along the centerline (Figure 10B). The PISP system (Philips IntelliSearch Portal v5.0.2.10010) was used to show the accurate puncture site, reference point, puncture route, puncture depth (113.0 mm), and drill depth (9.4 mm) by performing a CT scan of 1 mm for each layer before the surgery (Figures 10C,D). Cranial CT conducted the next day revealed the hematoma was almost completely evacuated (Figure 10E). MR diffusion tensor imaging showed that the drainage tube was placed beside the corticospinal tract to remove the hematoma, quickly relieving the pressure on the corticospinal tract, promoting the recovery of the limbs (Figure 10F).

## Discussion

The role of minimally invasive surgery, such as neuroendoscopic evacuation or stereotactic aspiration, has gained wide acceptance for the treatment of ICH in recent years owing to the benefit of surgical clot evacuation with less tissue damage, a shorter time required for surgery, and an increased possibility of using local anesthesia (20–22). A multicenter study involving 135 hospitals, including 841 patients with a basal ganglia hemorrhage, demonstrated that puncture suction is the optimal treatment when the volume of the hematoma was <50 ml (23). However, localization of the hematoma is always based on the experience of the surgeons, according to most published studies of endoscopic hematoma evacuation, causing instability, unreliability, and issues with quality control (24, 25). Although hematoma localization could be based on neuronavigation, which can accurately locate the desired position, the thinnest scan layers of CT data are necessary (26). In addition, there is a drawback to neuronavigation due to its time-consuming preparation (27). Therefore, the debate remains regarding how to select an adequate method of hematoma localization to obtain a mass proportion effect (28, 29). Here, we show our single-center experience with applying an easy, fast, and economical brain surgery head frame combined with a location sticker for the removal of hematomas from 45 consecutive patients with ICH.

Because the hematoma was removed through puncture, accurately guiding the drainage tube to the center of the hematoma is the key to optimal evacuation. Using our method, the operator could successfully locate the body of the hematoma and quickly introduce the drainage tube to the pre-established point by using this easy, fast, effective, and economical procedure. Therefore, the amplitude of swinging of the puncture tube was reduced, and procedure-related adverse effects on brain tissue were also minimized. The time required for preoperative surgical planning was ~5 min, and the hematoma evacuation time was 20–40 min depending on the number of required tubes. Wound closure is not necessary for our procedure. Thus, the total procedure time was <40 min. Concerning the optimal start time, traditionally, early surgery can improve the prognosis *via* decreasing secondary insults, however, achieve a lower hematoma evacuation rate. Jang et al. (30) performed a study involving 35 patients to compare the aspirated volume during surgery based on the surgical timing from symptom onset. Surgery after 120 h revealed the highest efficiency in terms of hematoma aspiration compared with the time period within 12 h, between 12 and 24 h and between 24 and 120 h. Additionally, the STICH II trial uncovered that patient undergoing an operation before 21 h from ictus had a trend toward getting a better outcome (29). Therefore, the optimal start time plays a key role in affecting the rebleeding risks and secondary brain injuries. In this study, the average time from symptom onset to hematoma aspiration was 9.356 h (at least 6 h and at most 16 h). Similarly, Tang et al. (31) performed a meta-analysis revealed that, within 24 h, minimal invasive surgery was associated with a low mortality rate and rebleeding rate, as well as a significant improvement of the prognosis and the quality life of patients when compared with conservative medical treatment or craniotomy. The amount of intraoperative blood loss was within 10 ml in all cases owing to the small procedural wound and less brain tissue injury, achieving a mean evacuation rate of 72.20%. Simultaneously, the average GCS score at 1 week after surgery and at the time of hospital discharge were 11.55 ± 2.59 and 12.86 ± 2.04, and the muscle power was 2.20 ± 1.64 and 2.88 ± 1.64, respectively, which shows a statistically significant improvement compared with preoperational.

Although stereotaxic devices are acceptable for the puncture of intracerebral hematoma, there are many disadvantages to the procedure. The installation of the framework of the stereotactic apparatus is necessary before the operation by screwing the head nails into the skull, causing significant pain. General anesthesia or basic intravenous anesthesia is always unavoidable because patients with cerebral hemorrhage usually appear restless and have difficulty cooperating with the installation process. CT data should be imported into the computer workstation to make an operation plan after performing a thin slice CT scan, which extends the whole operation time. In contrast, the installation of an apparatus is not necessary before the operation in our study, and the preoperative surgical planning can be completed quickly and accurately by using the PISP system, which uses the work station of the CT machine and could precisely locate the puncture route, puncture depth, and drill depth to avoid important brain functional areas, large blood vessels, and the sinus, and it is available in most of the hospitals.

Multiple types of targeting and puncture routes could be designed with the assistance of a brain surgery head frame and a location sticker to select the best operative plan based on the different locations and volumes of the hematoma. For hematomas in the basal ganglion region, high-occipital puncture along the long axis of hematoma is frequently used. Placing the lateral pore of the drainage tube at the center to aspirate, dissolve and drain the hematoma, then eliminating the compression to the corticospinal tract can preserve the neurological functions.A high-occipital puncture route is a safe way to drain the thalamic hemorrhage because it avoids functioning regions and large blood vessels. For cerebellar and brain stem hematoma, an accurate puncture could avoid craniotomy to reduce the operative time and trauma. For lobe hemorrhage, the route of puncturing is different based on the exact location. For large volumes of intracranial hematoma, multiple punctures from different layers could be performed. The drainage tube in the lower layer is placed in the front, while the high layer is placed at the back to aspirate the hematoma simultaneously. The gravity effect could drive the liquefied hematoma to flow into the lower tube after injecting urokinase so that the hematoma could be eliminated.

In this study, the drainage tube was soft with a blunt tip and a large lateral pore. The blunt tip of the drainage tube could push the encountering vessels aside without cutting the vessels during the puncture. The tube could move during the restoration of the brain tissue without cutting it during the hematoma reduction.

Our research also has some limitations that should be considered. Although the results of using a brain surgery head frame and location sticker combined with urokinase infusion for spontaneous ICH seems encouraging, the number of patients involved in our study was limited and it lacked a control group. A prospective randomized controlled study is necessary to compare the safety and effectiveness of this approach with the conventional aspiration of spontaneous ICH.

## Conclusion

Using this brain surgery head frame and location sticker combined with urokinase infusion appears simple, safe, and effective for the removal of hematoma from patients with spontaneous ICH. However, RCTs are necessary to provide more concrete evidence-based results.

## Data Availability Statement

The raw data supporting the conclusions of this article will be made available by the authors, without undue reservation.

## Ethics Statement

The studies involving human participants were reviewed and approved by the Medical Ethical Committee of Tangshan Gongren Hospital. The patients/participants provided their written informed consent to participate in this study. Written informed consent was obtained from the individual(s) for the publication of any potentially identifiable images or data included in this article.

## Author Contributions

HW, WX, and JC designed the study, acquired the data and drafted the article, analyzed and interpreted the data, and revised the article critically for important intellectual content together. All authors contributed to the article and approved the submitted version.

## Funding

This work was supported by Multi-center Real World Study on minimally invasive Treatment of Hypertensive Intracerebral hemorrhage guided by brain surgery head frame (GN-2020R0010).

## Conflict of Interest

The authors declare that the research was conducted in the absence of any commercial or financial relationships that could be construed as a potential conflict of interest.

## Publisher's Note

All claims expressed in this article are solely those of the authors and do not necessarily represent those of their affiliated organizations, or those of the publisher, the editors and the reviewers. Any product that may be evaluated in this article, or claim that may be made by its manufacturer, is not guaranteed or endorsed by the publisher.
